# Supplementary figures and images for: Interactions between copper homeostasis and the fungal cell wall affect copper stress resistance
Source: PLoS Pathog. 2022 Jun 23;18(6):e1010195. doi: 10.1371/journal.ppat.1010195 (PMC9258870; doi:10.1371/journal.ppat.1010195)

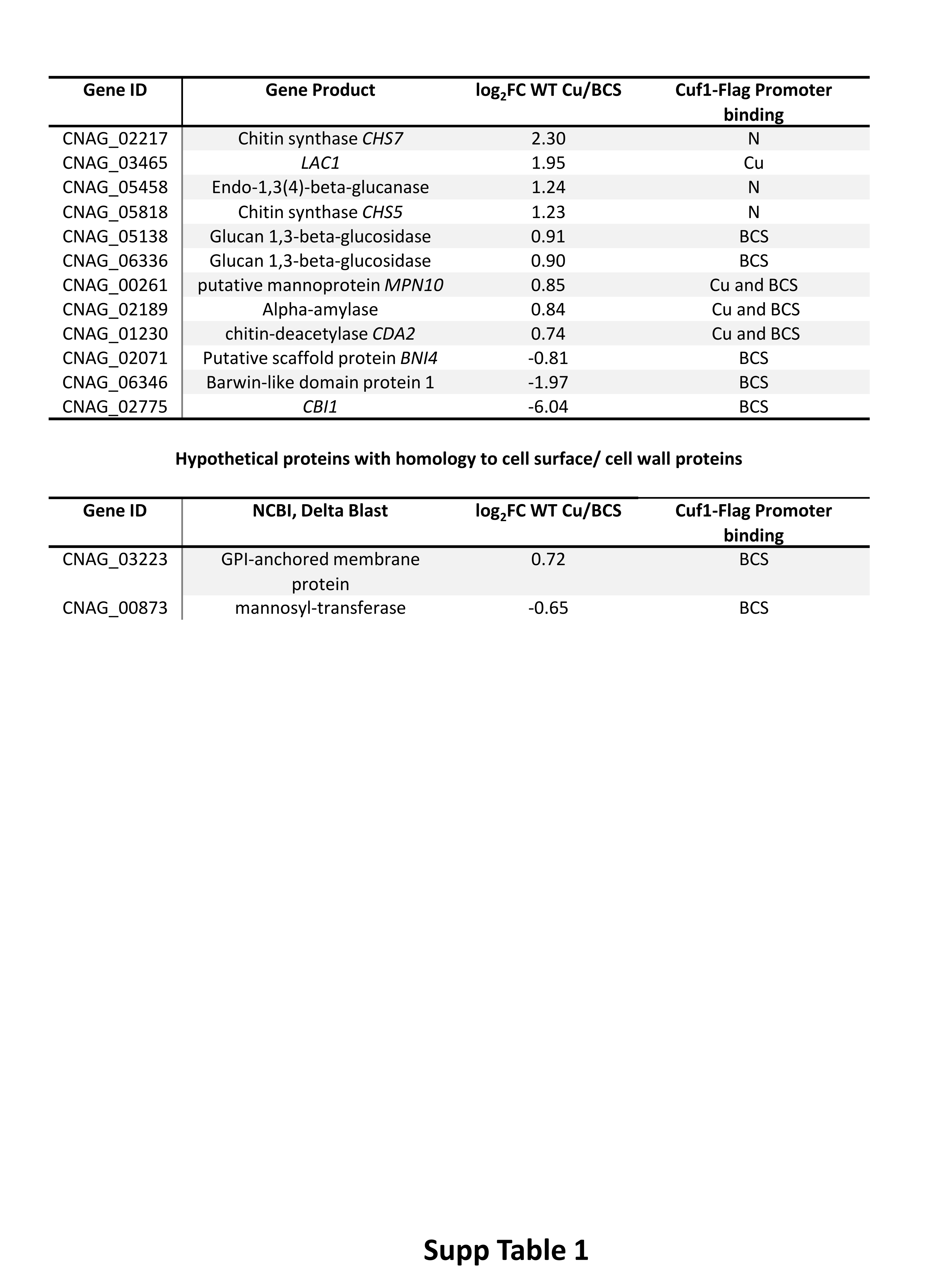

Supplement: S1 Table — Shown is the Gene identifier, predicted gene product, and the log2 fold change in high Cu-treated WT versus low Cu-treated WT cells (log2FC WT Cu/BCS). Cuf1-Flag promoter binding is indicated as follows: N = No binding, Cu = Binding during high copper stress, BCS = Binding during low copper stress, Cu and BCS = Binding during both copper stress conditions. (TIF) [file ppat.1010195.s001.tif]

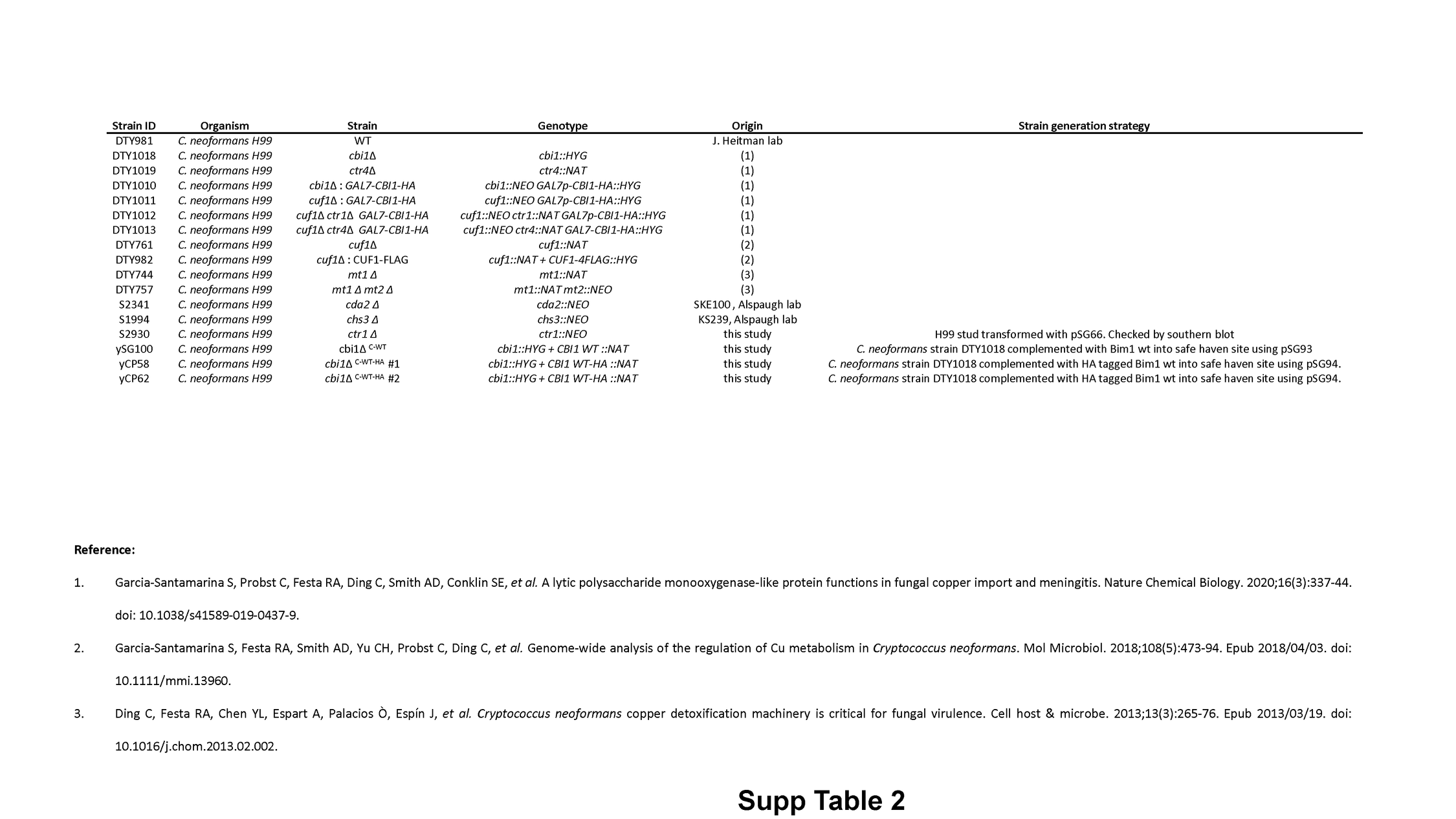

Supplement: S2 Table — (TIF) [file ppat.1010195.s002.tif]

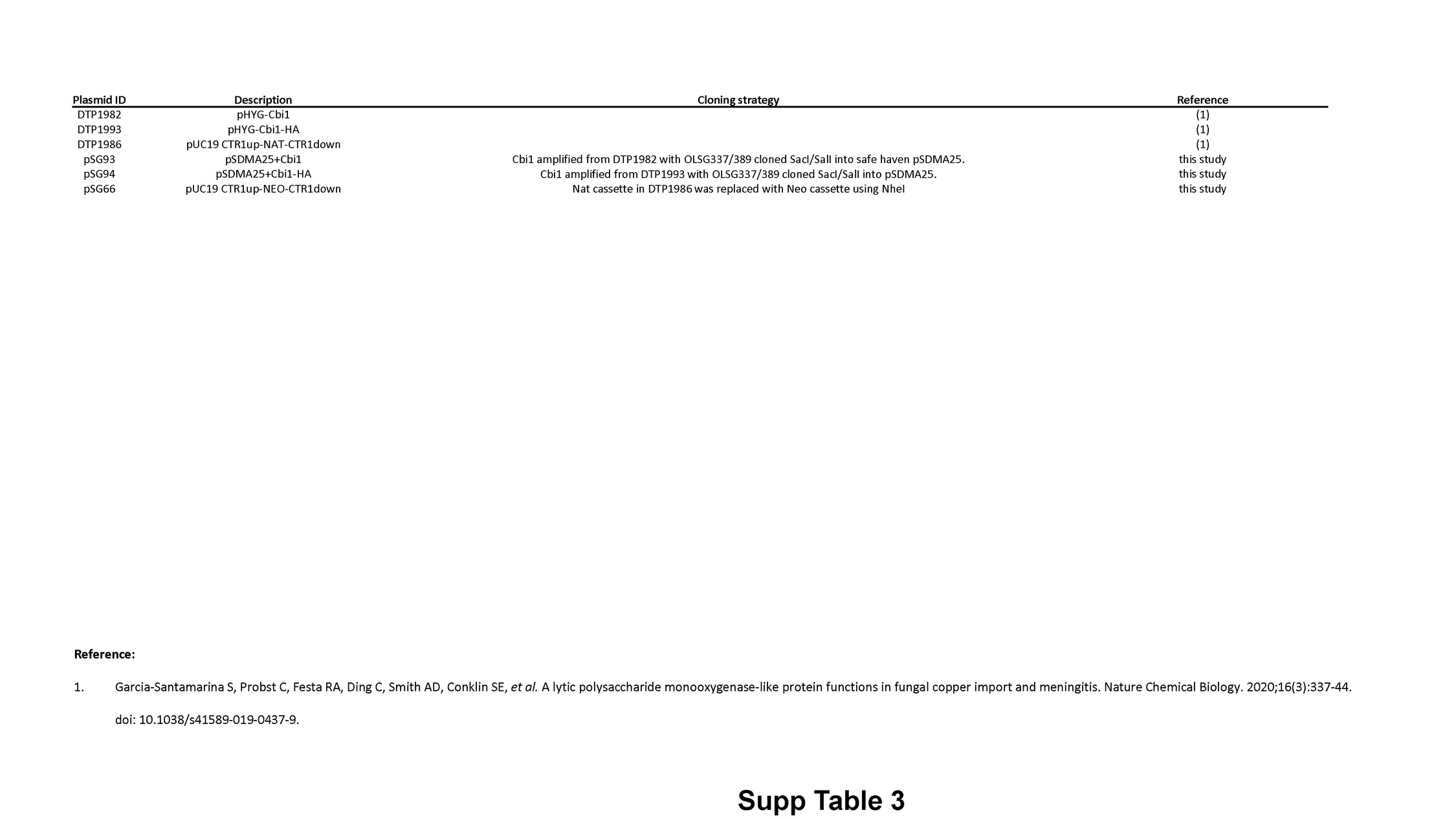

Supplement: S3 Table — (TIF) [file ppat.1010195.s003.tif]

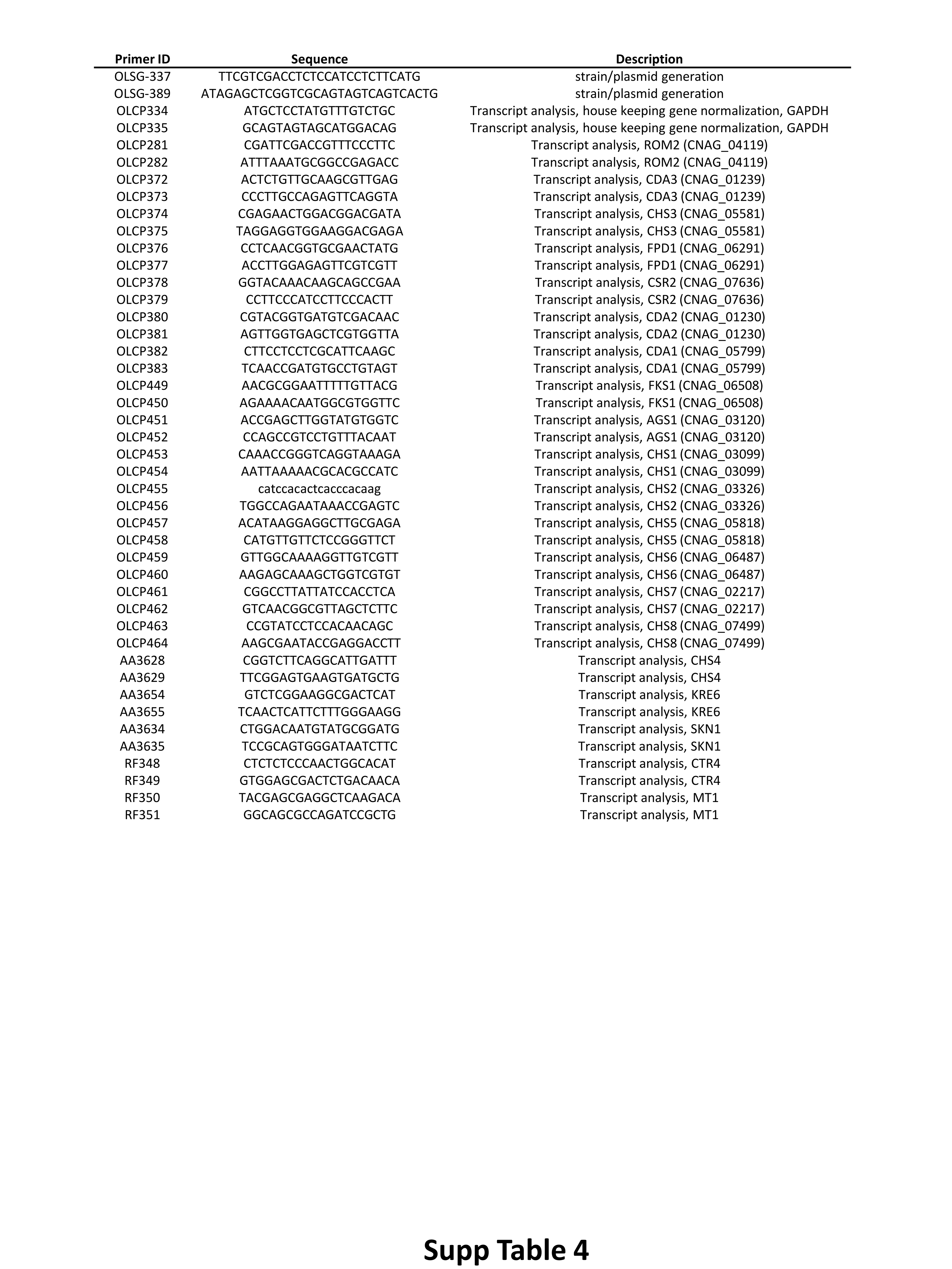

Supplement: S4 Table — (TIF) [file ppat.1010195.s004.tif]

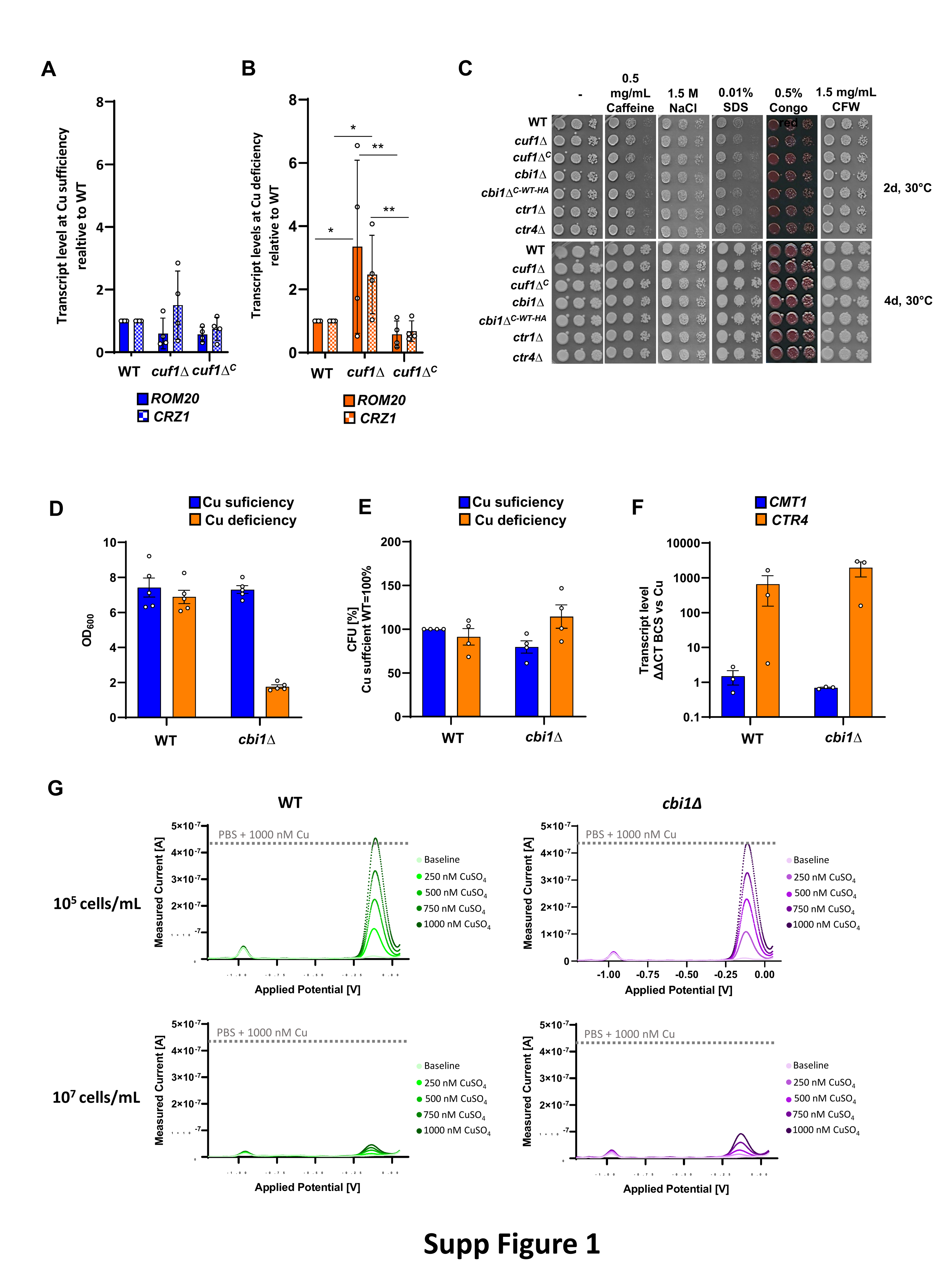

Supplement: S1 Fig — (A-B) qRT-PCR analysis of the ROM20 and CRZ1 transcript level in indicated strains during high copper (A) and low copper (B) stress. For the high copper condition, the WT, cuf1Δ and Cuf1-Flag complemented cuf1ΔC strains were inoculated to OD600 0.3 in SC supplemented with 1 mM CuSO4 and cultivated for 1h at 30°C. To induce low copper conditions, indicated strains were inoculated to OD600 0.3 in SC supplemented with 1 mM BCS and cultivated for 6h at 30°C. For comparison the WT transcript levels at each condition were set to 1. Presented is the mean +/- SEM of the relative transcript levels of 4 biological replicates. A 2-way ANOVA was performed using GraphPad Prism from log transformed data. (C) Growth analysis in presence of cell wall/ surface stressors. The spotting assay was performed on SC supplemented with indicated amounts of cell wall and cell surface stressors. Indicated strains were grown overnight in SC at 30°C. Cells were diluted to OD600 of 0.25 and a serial 1:10 dilution was spotted on to media plates. Plates were incubated at 30°C for 2-4d. This figure shows a representative image from 3 independent spotting experiments. (D) Growth rate of copper sufficient or deficient WT and cbi1Δ cells. Cells were incubated in YPD supplemented with 10 μM CuSO4 (Cu sufficiency) or with 250 μM BCS (Cu deficiency) for 24h at 30°C. Growth was measured through 0D600. Presented is the average +/- SEM of 5 biological replicates. (E) Colony forming unit (CFU) analysis of copper sufficient or deficient WT and cbi1Δ cells. Cells were treated as described in (B). After 24h of growth, cells were diluted to OD600 1. 200 μL of a serial 1:1000 dilution were plated onto YPD plates and colonies were counted after 3d of incubation at 30°C. The CFU of copper sufficient WT was set to 100%. Presented is the average +/- SEM of the relative CFU (as compared to copper sufficient WT) from 4 biological replicates. (F) qRT-PCR analysis using CMT1 and CTR4 as indicator for Cu toxicity or defici [file ppat.1010195.s005.tif]

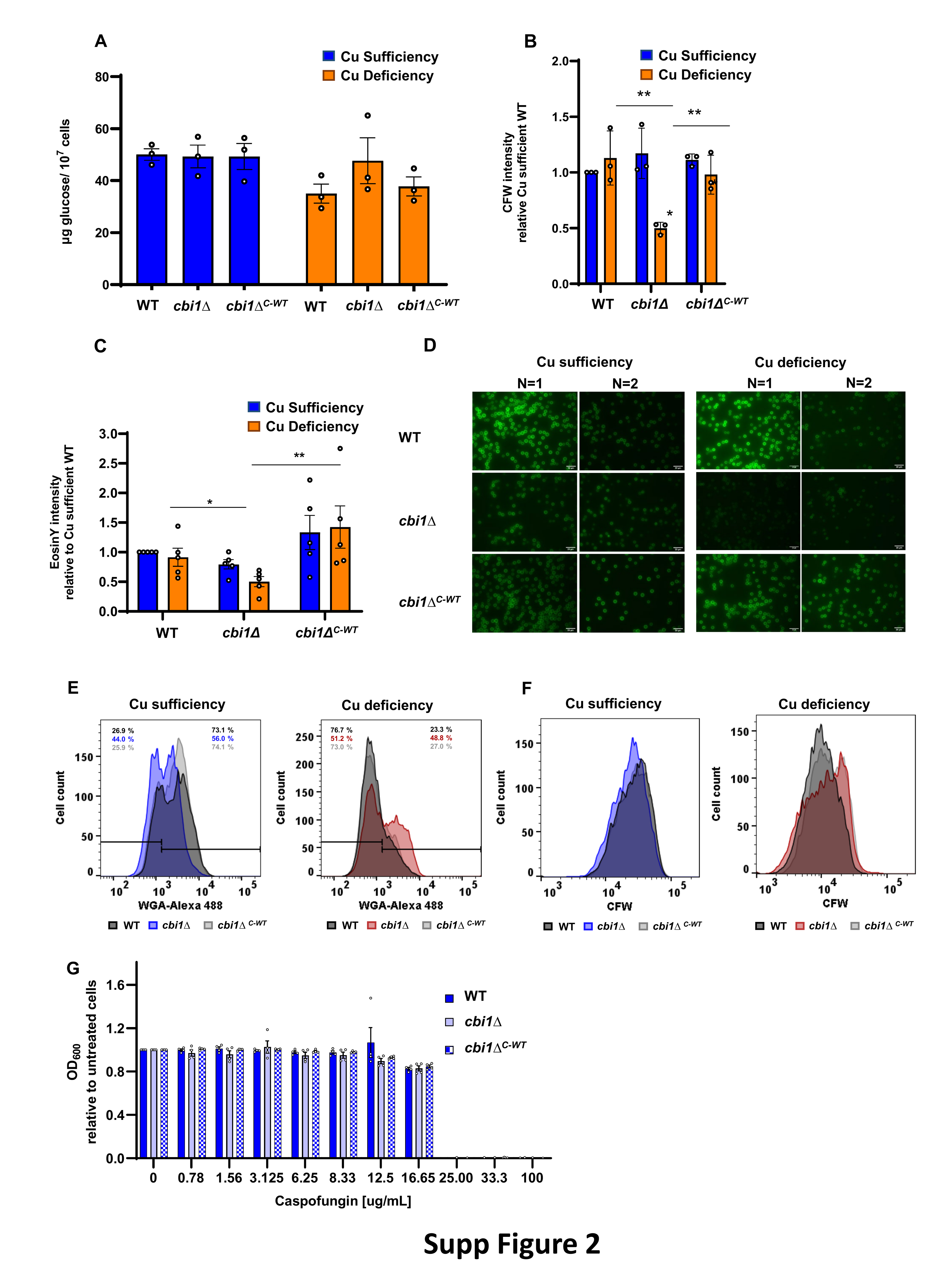

Supplement: S2 Fig — (A) β-glucan quantification of copper sufficient and copper deficient wt, cbi1Δ and cbi1ΔC-WT complemented cells. Strains were incubated for 24h in YPD+ 10 uM CuSO4 (Cu sufficiency) or YPD +250 uM BCS (Cu deficiency) and then harvested, cell counted and lyophilized. The Megazyme yeast b-glucan kit was used for quantification of b-glucan from lyophilized cells. Values are shown in ug Glucose / 107 cells. Presented is the average +/- SEM of 3 biological replicates. (B) Calcofluor white (CFW) and wheat germ agglutin (WGA)-Alexa 488 staining for chitin of copper sufficient or deficient WT, cbi1Δ and and Cbi1 WT complemented cbi1Δ (cbi1ΔC-WT) cells. Strains were cultivated as described in (A). Shown is the mean +/- SEM of the relative CFW intensity from 3 independent experiments. The CFW intensities were measured with ImageJ/Fiji and normalized to cell count. Shown is the relative CFW intensity (copper sufficient WT set to 1). A 1-way ANOVA was performed using GraphPad Prism from log transformed data. (C) EosinY staining for chitosan of copper sufficient and deficient WT, cbi1Δ and cbi1ΔC-WT complemented cells. Strains were cultivated as described in (A), followed by EosinY staining. Shown are representative images for 2 two independent experiments. Five independent treatments and stainings were performed. (D) Relative EosinY intensity from 5 independent experiments. The EosinY intensities were measure with ImageJ/Fiji and normalized to cell count. Shown is mean +/- SEM of the relative EosinY intensity (copper sufficient WT set to 1). A 1-way ANOVA was performed using GraphPad Prism from log transformed data. (E-F) FACS analysis of CFW and WGA-Alexa 488 stained cells. WT, cbi1Δ and cbi1ΔC-WT complemented cells were cultivated as described in (A) (E) WGA-Alexa 488 staining histogram representation of the FACS analysis depicted in Fig 3. (F) CFW-staining histogram representation of FACS analysis depicted in Fig 3. (G) Minimal inhibitory concentration (MIC) analysis of Casp [file ppat.1010195.s006.tif]
